# p53 binding to human genome: crowd control navigation in chromatin context

**DOI:** 10.3389/fgene.2014.00447

**Published:** 2014-12-22

**Authors:** Krassimira Botcheva

**Affiliations:** Cell and Molecular Biology Department, Life Sciences Division, Lawrence Berkeley National Laboratory, Berkeley, CA, USA

**Keywords:** p53 tumor suppressor network, genome-wide binding, epigenetic regulation, chromatin, CpG islands, repeats, navigation

## Abstract

p53 is the most studied human protein because of its role in maintaining genomic stability. Binding to genomic targets is essential for transcription-dependent p53 tumor suppression, but how p53 selects targets remains unclear. Here, the impact of chromatin context on p53 genome-wide binding and targets selection is discussed. It is proposed that p53 genomic binding serves not only to regulate transcription, but to sense epigenomic changes threatening the genomic integrity. The problem of p53 navigating the human genome is discussed with respect to the degenerate p53 binding motif. This discussion relates to the fundamental problem of DNA binding factors navigating large genomes in search for cognate binding sites.

## 35 YEARS p53

p53 guards the genome by mobilizing a complex network of target genes and coordinating the cellular stress responses to prevent propagation of damaged DNA (Vogelstein et al., 2000). DNA binding is important for the p53 tumor suppression; 90% of the cancer-associated p53 mutations reside in the DNA binding domain (Hollstein et al., 1991). It is commonly viewed that in absence of stress cells keep low amount of p53, bound to some genomic targets without affecting transcription, while upon DNA damage activation, the genomic binding is accompanied by transcription regulation. Depending on the cell type and damage, p53 regulates different sets of genes, triggering cell cycle arrest, DNA repair, senescence, or apoptosis, steering the cells toward life or death (Vousden and Lu, 2002). How p53 selects its targets remains one of the foremost questions in the field.

Binding to DNA and transcription regulation are affected by the *p53 mutational load* (Leroy et al., 2014), extensive *posttranslational modifications* (Meek and Anderson, 2009), *tertiary structure and oligomerization state* (Joerger and Fersht, 2010), *binding partners and cooperativity* (Braithwaite et al., 2006; Brandt et al., 2012), *basal transcription machinery* (Espinosa, 2008), and *genomic binding sites* (Horvath et al., 2007; Riley et al., 2008; Wang et al., 2009). Adding to this complexity, the *family members p63 and p73* (Belyi et al., 2010) and the *isoforms expression patterns* in the family (Bourdon et al., 2005; Marcel et al., 2014) have a major impact on the p53 properties, collectively defining functions previously ascribed to a single full length protein. While these components are undoubtedly important for the p53 genomic binding and transcription regulation, they function in the context of *chromatin,* which plays an active role in transcription and moderates the interactions with DNA (Li et al., 2007).

## p53 NETWORK COMPLEXITY

**FIGURE 1:**
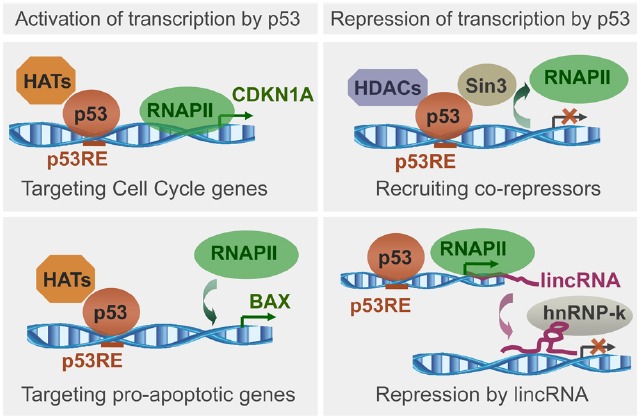
**Schematic representation of p53 ability to load histone modifiers upon binding to genomic targets, followed by recruitment or exclusion of RNA polymerase II (RNAPII), and transcription regulation.** Illustrated is transcription activation upon loading of histone acetyltransferases (HATs) to promoters of genes regulating cell cycle or apoptosis, and transcription repression upon loading of histone deacetylases (HDACs), or through lincRNAs. Based on recent reviews (Beckerman and Prives, 2010; Rinn and Huarte, 2011).

The p53 ability to load histone modifiers and chromatin remodelers upon binding to genomic targets is essential for the transcription regulation (Figure 1) and has been well documented (Beckerman and Prives, 2010; Rinn and Huarte, 2011). The opposite, the effect of the *chromatin context on p53 targets selectivity* is far less understood. Just as transcription factor binding affects the local chromatin, the chromatin context affects the transcription factor binding (Guertin and Lis, 2013). Genomic binding dependent on the chromatin context has been reported for proteins such as Myc (Guccione et al., 2006) and NF*κ*B (Natoli, 2009). The chromatin state matters because it is plastic and dynamic, changing during normal development (Mikkelsen et al., 2007) and in cancer (Baylin and Jones, 2011). The chromatin context introduces a level of complexity in the p53 network, which needs to be considered to understand the p53 functions dependent on DNA binding. Even in a “competent state” for sequence-specific DNA binding (without mutations, activated by posttranslational modifications), p53 may not bind to sites buried in inaccessible chromatin, and bind if they become available. Despite the proposed and observed chromatin impact on p53 binding (Lidor Nili et al., 2010; Millau et al., 2011), there is no good understanding of how p53 binding sites availability is defined and how it affects the genomic targets selection. Analysis of the binding context relies on the binding sites detection.

## p53 GENOMIC BINDING SITES

Broadly, two types of experiments provide binding sites information, individual gene studies and genome-wide studies. Evidence for p53-occupied genomic sites came first from individual gene studies. About 200 such sites were reported (Horvath et al., 2007; Riley et al., 2008; Wang et al., 2009). Hereafter these are called reference REs (response elements), since p53 binding at them was demonstrated to evoke transcriptional response. Development of the sequencing technologies enabled genome-wide studies at unprecedented depth, allowing mapping of global binding patterns and chromatin state analysis (Park, 2009; Landt et al., 2012), including large scale p53 binding studies.

Since the discovery of the p53 consensus motif RRRCWWGYYY-_N(0–13)_-RRRCWWGYYY (El-Deiry et al., 1992; Funk et al., 1992), efforts were focused on designing methods for robust detection of p53 occupied sites. Early work (Tokino et al., 1994), followed by limited scale studies (Cawley et al., 2004; Chen and Sadowski, 2005; Hearnes et al., 2005; Kaneshiro et al., 2007), generated data allowing extrapolation to the whole human genome. The estimated 1,500–3,500 sites occupied genome-wide, were in agreement with predictions by computational models (Veprintsev and Fersht, 2008). The first high-throughput p53 ChIP-PET approach based on Sanger and 454 sequencing (Ng et al., 2006; Wei et al., 2006) paved the road for the next-generation sequencing p53 studies. Most of them used ChIP-seq to map *endogenous wild type p53* genomic binding in *human cell lines* (Botcheva et al., 2011; Smeenk et al., 2011; Nikulenkov et al., 2012; Menendez et al., 2013; Akdemir et al., 2014; McDade et al., 2014; Rashi-Elkeles et al., 2014); some mapped p53 *family members*, p53 *mutants*, or p53 *variants* (Kouwenhoven et al., 2010; Koeppel et al., 2011; Martynova et al., 2012; Schlereth et al., 2013; Wang et al., 2014); others looked at p53 binding in the *mouse genome* (Li et al., 2012; Kenzelmann Broz et al., 2013), and recently p53 binding data were generated by ChIP-exo (Chang et al., 2014).

## DEFAULT AND DISTINCT BINDING

What these studies revealed about the *endogenous wild type p53 binding to the human genome*? Genomic binding is not always followed by transcription changes; p53 exhibits both “default” and “distinct” binding. The studies coupled with expression analysis agreed that many p53 occupied sites are not associated with transcription changes (Li et al., 2012; Nikulenkov et al., 2012; Kenzelmann Broz et al., 2013; Menendez et al., 2013; Akdemir et al., 2014; McDade et al., 2014; Rashi-Elkeles et al., 2014), which could be interpreted as absence of transcription regulation at these sites. Since p53 has induced and constitutive functions, and binding may serve to induce transcription, but also to maintain basal transcription levels (Zheltukhin and Chumakov, 2010), p53 occupancy at some of these sites may support the basal expression of the nearby genes. Although not manifested by impressive transcription changes, maintaining global gene expression patterns, ensuring functional homeostasis under stress, in the damaged genome, may represent important p53 function. Since p53 binds to enhancers (Melo et al., 2013), occupancy at some of these sites may exert long distance effect on gene expression.

p53 ability to induce stress and cell type specific responses is well recognized, but whether p53 binds to its targets in such selective manner has been a subject of debate. Recent genome-wide binding studies revealed common and distinct binding patterns. Interestingly, treatment with Nutlin (small molecule activator of p53) leads to more p53 binding, but less effect on transcription, compared to DNA damage (Nikulenkov et al., 2012; Menendez et al., 2013). A “p53 default program” was proposed to explain the observation that the most frequently occupied p53 sites were the same after different treatments (Nikulenkov et al., 2012). Besides the detected p53 at common sites, distinct binding patterns were observed as well (Botcheva et al., 2011; Menendez et al., 2013; Akdemir et al., 2014).

The proposed “p53 default program” can explain the occupancy at canonical targets (such as CDKN1A and MDM2), consistently reported bound under many stress conditions and cell types analyzed. On the other hand, significant fraction of the binding sites displays occupancy dependent on the type of treatment (Menendez et al., 2013; Akdemir et al., 2014), or on the cell context (Botcheva et al., 2011; Botcheva and McCorkle, 2014). If the “default” binding sites are strong, unambiguously detected by many, and reported “functional,” therefore clearly important for the transcription regulation, why obsessing with the “distinct” binding patterns, which despite being shaped by a large fraction of the data, may be composed by sites with less well defined individual contribution to direct transcription regulation? The “default” binding sites, being consistently occupied, may reflect fundamental p53 functions important in any cell context or stress type, while the distinct binding patterns may reveal more about the stress- and cell type- specific p53 functions. Patterns matter in nature. For example, despite the importance of individual populations for biodiversity, gross changes in tropical forests have major impact on earth climate (Lewis, 2006). In order not to lose the forest for the trees, it may be worth studying the global patterns associated with p53 binding, since these may reflect global trends in the binding context, affecting the way p53 network is engaged in response to stress.

## THE CHROMATIN CONTEXT

What causes the distinct genomic binding patterns of the *endogenous wild type p53*? Cancer-derived human cell lines have been widely used for p53 research (Millau et al., 2010). When high-resolution p53 binding map was generated for the first time in normal, not immortalized human cell line (Botcheva et al., 2011) and compared to previous studies in cancer cell lines (Wei et al., 2006; Smeenk et al., 2008, 2011), distinct p53 binding patterns were observed. Only in the normal cells p53 was strongly enriched within 2 kb of transcription start sites and at CpG islands (CGIs); distribution typical for the functional reference REs, but not for the sites mapped in the cancer cell lines, where p53 was depleted from CGIs. The chromatin structure at CGIs is important for the regulation of transcription; these CG rich sequences are kept hypomethylated in otherwise methylated genome, and subjected to epigenetic control (Deaton and Bird, 2011). Correlating p53 binding with high-resolution methylome generated in the same cell line (Lister et al., 2009), revealed enrichment of p53 binding sites at hypomethylated DNA. Importantly, that was true not only for sites in CGIs (generally kept hypomethylated), but for sites out of CGIs as well (Botcheva et al., 2011). Notably, when p53 DNA binding in methylation-dependent mode was first considered and examined, *no specific p53 affinity* was detected for particular methylation state of the binding *sites on naked DNA* (Petrovich and Veprintsev, 2009). Thus, p53 propensity to bind at hypomethylated regions (not just CGIs) was likely due to the chromatin structure modulated by the DNA hypomethylation, rather than affinity to hypomethylated binding site (Botcheva et al., 2011), although at present that could not be excluded.

Due to the nature of the distinct p53 binding patterns (enrichment at hypomethylated DNA and CGIs in the normal cell line; depletion from CGIs and higher enrichment at repeats in the cancer cell lines), it was proposed that epigenetic changes accompanying cancer progression (local CGIs hypermethylation and global genomic hypomethylation) modulate p53 binding to the genome (Botcheva et al., 2011). Another possibility suggested that at certain sites, the methylated CpGs may undergo tumorogenesis-dependent deamination, to eliminate p53 responsiveness (Freed-Pastor and Prives, 2011). Subsequently, a key study in mouse fibroblasts revealed that in absence of wild type p53, DNA demethylation triggers repeats instability, followed by massive apoptotic response (Leonova et al., 2013), commented in details (Levine and Greenbaum, 2012; Koonin, 2013; Tavana and Gu, 2013). These findings have implications beyond gene promoters and repeats, because cancer is accompanied by global epigenetic alterations involving enhancers and insulator elements as well (Taberlay et al., 2014), and binding at enhancers is important for the p53-dependent transcription regulation.

The datasets analyzed by Botcheva et al. (2011) differed not only by cell context (normal and cancer-derived cell lines), but by treatment and/or experimental approach, potentially contributing to the observed p53 binding differences. Recently, the distinct p53 binding patterns were confirmed under same treatment, experimental conditions and ChIP-seq approach, evidence for cell context-dependent p53 genomic binding (Botcheva and McCorkle, 2014). Moreover, the analysis was extended to examine the differentially bound types of repeats and p53 was found enriched at LINE (long interspersed nuclear elements) in the cancer cell line HCT116, compared to the normal IMR90 (Botcheva and McCorkle, 2014). Epigenetic dysregulation at repeats is a major cancer landmark, and hypomethylated LINE repeats (normally methylated) are associated with bad colon cancer prognosis (Ogino et al., 2008); thus, p53 enrichment at LINE repeats in the colorectal cell line HCT116, might be due to cancer-associated LINE hypomethylation (Botcheva and McCorkle, 2014). The distinct p53 binding patterns in HCT116 and IMR90 likely reflect differences in the epigenetic landscapes in these cell lines, due to cancer-associated changes (accumulated in HCT116), overlaid on tissue-specific differences (HCT116 has epithelial, while IMR90 has mesenchymal origin; Botcheva and McCorkle, 2014). In the future, it would be very interesting to investigate the involvement of the p53 transcriptional network in the process of cancer-associated EMT (epithelial mesenchymal transition).

## SENSING THE EPIGENOME

**FIGURE 2:**
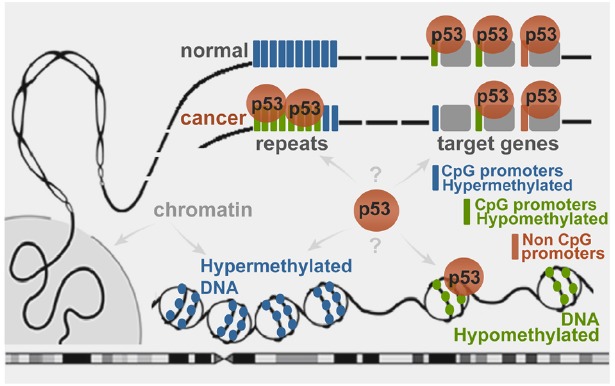
**Schematic representation of wild type p53 binding to genomic targets in a DNA methylation-dependent manner.** In normal cells the stress-induced p53 binds to sites residing in hypomethylated regions. Cancer-associated epigenetic changes may lead to local p53 depletion from hypermethylated target promoters and enrichment at globally hypomethylated regions, such as repeats (Botcheva et al., 2011; Botcheva and McCorkle, 2014).

The epigenetic impact on p53 genome-wide binding (Botcheva et al., 2011), and p53-dependent transcription regulation (Leonova et al., 2013) has far reaching implications. The p53 genomic targets selection is important for tumor suppression, yet the mechanisms are unclear. These studies suggest p53 genomic binding dependent on DNA methylation (Figure 2). While it is known that transcription factor binding can be affected by the methylation status of the CpGs in their binding sites (Chen et al., 2011), the probable mechanisms remain to be defined. Interestingly, although the OCT4 motif does not contain CpGs, in human embryonic stem cells this protein is excluded from target sites residing in DNA methylated locations (You et al., 2011; Jones, 2012).

p53 has been long known as a keeper of the genomic integrity, and *sensing the epigenome* might be important part of it. While searching for binding sites, p53 may be “screening” the genome for DNA hypomethylation at regions “normally” hypermethylated (residing in inaccessible chromatin context), which would become available for p53 binding upon hypomethylation. Global DNA hypomethylation on a large scale could expose numerous sites, causing global redistribution of the p53 binding and readjusting its transcriptional program to maintain genomic stability.

While “guarding the genome” does not involve direct recognition of genetic lesions by p53, sensing the epigenome may imply direct recognition of the regions of epigenetic changes. Moreover, the extensive network of p53 *binding sites at repeats* (Wang et al., 2007; Harris et al., 2009; Zemojtel et al., 2009; Cui et al., 2011; Botcheva and McCorkle, 2014) could serve as a system of sensors for detection of epigenetic perturbations, which could destabilize the repeats and the whole genome. Therefore, the reason for the existence of p53 binding sites at repeats may be monitoring for epigenetic aberrations. Upon *comparing p53 binding in cancer cell lines* under different stress, common global trends may emerge (besides the “default program”) that are dependent on the epigenetic changes, shared at least to some extent by these cell lines. Considering the magnitude of such epigenetic changes, the common trends could dominate the p53 binding landscape, obscuring the detection of more subtle stress-specific patterns and making the binding look “non-selective.”

p53 has been regularly reported bound to some of its genomic targets *in absence of stress*, often interpreted as allowing for fast transcriptional response upon stress induction. Considering the p53 binding at hypomethylated DNA, it is possible that p53 occupies constitutively some of its key targets to *protect them from hypermethylation*, which may make them inaccessible for p53 binding, abrogating its tumor suppression program.

Mammalian cells acquire epigenetic hallmarks of human cancer during immortalization (Tommasi et al., 2013). If p53 indeed is sensing the epigenome at the level of genomic binding, one may expect p53 *binding patterns in immortalized cells* to be more similar to those in cancer cells, rather than to those in normal cells. In support of that, ChIP-chip study examining p53 binding in different cell contexts demonstrated that immortalized fibroblasts behaved as cancer cell lines, unlike the normal cells (Shaked et al., 2008). Studying the changing p53 binding patterns during immortalization could reveal information about the epigenetic modulation of the p53 network functions early in tumorogenesis.

Do these findings have *clinical implications*? In certain contexts, wild type p53 may serve “oncogenic” functions and may not need further activation. Interestingly, there are colorectal cancer subgroups with CGI methylator phenotype (CIMP-high, CIMP-low) less likely to accumulate p53 mutations (Hinoue et al., 2012). It may be because they need wild type p53 to protect them from the deleterious effects of aberrant DNA methylation. In such cases, use of demethylating agents could be explored. One such drug, Decitabine, has been approved by FDA since 2006 for treating myelodysplastic syndromes, and has shown promising results on epithelial tumor cells as well (Tsai et al., 2012).

## CROWD CONTROL NAVIGATION

The high-confidence p53 binding sites annotated by a given genome-wide study represent only a small subset of the total sites detected; the vast majority of the sites are not associated with particular function. Considering the degenerate p53 consensus binding motif, computational models estimate hundreds of thousands sites in the human genome (Hoh et al., 2002; Veprintsev and Fersht, 2008). Why so many, if only few are functional? Degenerate binding motifs are often found for sequence specific DNA binding proteins in higher eukaryotes, unlike in prokaryotes (Kadonaga, 2004). Interestingly, despite the smaller genome, prokaryotic transcription factors have more specific binding motifs; despite the larger genomes, eukaryotic transcription factors have degenerate, lower specificity DNA binding motifs. Applying information theory to the genomic sites recognition by DNA binding proteins, minimum information content is required to specify unique position in the genome. Calculations of the average information content of prokaryotic and eukaryotic motifs (based on the motif length and the base frequency at particular positions) demonstrated that in prokaryotes single binding site is sufficient to address unique location in the genome, but not in eukaryotes (Wunderlich and Mirny, 2009). The average information content of a prokaryotic motif *I* ≈ 23 bits is slightly above the required *I*_min_ ≈ 22 bits to specify unique position in a prokaryotic genome (10^6^–10^7^ bp); the average information content of a multicellular eukaryotic motif *I* ≈ 12.1 bits is *far below* the minimum information required *I*_min_ ≈ 30 bits to specify unique position in eukaryotic genome of ∼10^9^ bp (Wunderlich and Mirny, 2009).

One may expect that eukaryotic DNA binding proteins, locating their sites in larger genomes, would have motifs with higher information content than prokaryotes. Instead it is the opposite. Because of motifs degeneracy, numerous DNA binding sites, besides the “functional” ones, are present for a given DNA binding protein (p53 including). Thus, “functionality” is specified by additional means, such as clustering of binding sites, combinatorial and cooperative binding (Kadonaga, 2004), and three-dimensional organization of the chromatin (Lieberman-Aiden et al., 2009; Edelman and Fraser, 2012; Amouyal, 2014). The point is, why on the first place, eukaryotic DNA binding proteins evolved to have degenerate motifs, meaning promiscuous binding to the genome, and requiring additional means to specify functionality? Why not evolving higher content information motifs which would specify unambiguously unique positions in the genome? It may be because the unique positions have to be not only specified, but found in the large genome.

The mode employed by eukaryotic DNA binding proteins to find their genomic sites may make sense from navigation point of view. According to current models for DNA binding sites search (Halford and Marko, 2004), and by p53 (McKinney et al., 2004; Tafvizi et al., 2011), a protein spends time “on” and “off” DNA, and “moving along DNA” is important (here, terms as “sliding” and “diffusing” are omitted since the point is not about how a protein propels on DNA, but how it navigates it). There are many DNA binding proteins presumably searching for their “functional” sites on DNA (Zabet and Adryan, 2013). It may look contra intuitive but the many degenerate binding sites, by transiently binding their cognate proteins, may actually serve the role to navigate and ensure “smooth traffic” on DNA, so that the “functional” sites could be reached efficiently (in everyday life, traffic lights and stop signs are placed in the most crowded traffic spots). Interestingly, “crowd control” theories have been long applied to animal kingdom and group decision making has been hypothesized to be beneficial in complex environments (Berdahl et al., 2013). It is possible that the DNA binding proteins in the large eukaryotic genomes rely on “crowd control navigation” provided by the multiple binding sites, in order to reach their “functional” binding sites and regulate transcription.

## CONCLUSION

The p53 network is perfectly fit to be interrogated by genome-wide approaches. That would allow otherwise hidden global patterns to be revealed and studied, to help interpreting the p53-dependent tumor suppression program in the context of dynamic chromatin and to dissect the interplay between genetic and epigenetic changes associated with cancer. The discoveries from the past 35 years expanded greatly the p53 knowledge and underlined the importance of addressing the p53 network complexity for the pathway’s full potential to be unlocked and applied in the clinic, because cancer progression and tumor suppression are two faces of the same coin and the p53 mark is undeniably stamped on both.

### Conflict of Interest Statement

The author declares that the research was conducted in the absence of any commercial or financial relationships that could be construed as a potential conflict of interest.
